# C-Series Coelenterazine-Driven Bioluminescence Signature Imaging

**DOI:** 10.3390/ijms232113047

**Published:** 2022-10-27

**Authors:** Genta Kamiya, Nobuo Kitada, Tadaomi Furuta, Takashi Hirano, Shojiro Maki, Sung Bae Kim

**Affiliations:** 1 Department of Engineering Science, Graduate School of Informatics and Engineering, The University of Electro-Communications, Chofu 182-8585, Japan; 2 School of Life Science and Technology, Tokyo Institute of Technology, B-62 4259 Nagatsuta-cho, Midori-ku, Yokohama 226-8501, Japan; 3 Research Institute for Environmental Management Technology, National Institute of Advanced Industrial Science and Technology (AIST), 16-1 Onogawa, Tsukuba 305-8569, Japan

**Keywords:** bioluminescence, signature, intensity pattern, coelenterazine, luciferase, reporter

## Abstract

The present study introduces a unique BL signature imaging system with novel CTZ analogues named “C-series.” Nine kinds of C-series CTZ analogues were first synthesized, and BL intensity patterns and spectra were then examined according to the marine luciferases. The results show that the four CTZ analogues named C3, C4, C6, and C7, individually or collectively luminesce with completely distinctive BL spectral signatures and intensity patterns according to the luciferases: *Renilla* luciferase (RLuc), NanoLuc, and artificial luciferase (ALuc). The signatural reporters were multiplexed into a multi-reporter system comprising RLuc8.6-535SG and ALuc16. The usefulness of the signatural reporters was further determined with a multi-probe system that consists of two single-chain probes embedding RLuc8 and ALuc23. This study is a great addition to the study of conventional bioassays with a unique methodology, and for the specification of each signal in a single- or multi-reporter system using unique BL signatures and patterns of reporter luciferases.

## 1. Introduction

Bioluminescence (BL) has been widely used as an efficient optical readout. BL is exhibited by a myriad of biological species, including fungi, beetle, and marine organisms [1]. Many BL systems consist of a catalytic reaction of luciferase with the substrate luciferin in the presence of cofactors. For example, D-luciferin (LH_2_) is catalyzed by beetle luciferases such as firefly luciferase (FLuc) and click beetle luciferase (CBLuc) in the presence of ATP and molecular oxygen (O_2_), and Mg^2+^ is used to generate green–red light (λ_max_ = 560~640 nm) [2,3]. On the other hand, the natural luciferin of marine organisms, native coelenterazine (nCTZ), is oxidized by corresponding luciferases such as *Gaussia* luciferase (GLuc), *Renilla reniformis* luciferase (RLuc), and *Oplophorus* luciferase (OLuc), in the presence of O_2_ and emits blue light (λ_max_ = 450~560 nm) [2,4].

Many luciferins have been developed through chemical modifications of the imidazopyrazinone backbone of nCTZ for use in the BL system. For instance, furimazine accounts for the majority of BL systems through a pairing with its specific luciferase, NanoLuc, as a small subunit of the OLuc [5]. This furimazine–NanoLuc pair has been applied in several BL systems, including a protein–fragment complementation assay (PCA) [6], bioluminescence resonance energy transfer (BRET) [7,8], and reporter gene assay [9].

Many CTZ analogues have developed luminesce with RLuc and its mutants, which emit various wavelengths of BL, ranging from 400 to 560 nm [4]. Some CTZ analogues have been designed to emit a broader color palette, covering blue to near infrared [10].

Some CTZ analogues have been applied not only to natural marine luciferases but also to artificial luciferases (ALucs); these were developed by extracting frequently occurring amino acids from the alignment of copepod luciferases [11,12]. This CTZ–ALuc imaging system was used to develop a series of single-chain probes to visualize the activities of endogenous hormones and bioactive small molecules [3]. 

Regarding their applications, multi-reporter imaging presents a key breakthrough in addressing the complexity of molecular events in living subjects. Nevertheless, most spectra of conventional reporter luciferases have broad bandwidths and are superimposed on each other. This optical contamination fundamentally cannot be overcome by spectral unmixing algorithms [13], optical filters [14], and quenching reagents [15]. 

This issue was partly addressed by Nishihara et al., who developed luciferase-specific substrates for selectively illuminating a reporter luciferase among multiple luciferases [16,17]. Specific tagging systems, such as HaloTag, have the potential to address such an optical contamination among multi-reporters [18]. Multiplex reporter gene assay systems may provide a solution to optical contamination between reporters if the transcription machinery, along with the optical filters, is strictly controllable [19].

In the present study, a unique BL signature imaging system with novel CTZ analogues named “C-series” is introduced. Nine kinds of the C-series CTZ analogues were first synthesized, and then the BL intensity patterns and specific spectra were examined according to the luciferases. The results show that the four CTZ analogues, namely, **C3**, **C4**, **C6**, and **C7**, individually or collectively luminesced with completely distinctive BL spectral signatures and intensity patterns, according to the major marine luciferases: RLuc, NanoLuc, and ALuc. 

The quantitative properties in reporter mixtures were determined with a multi-reporter system comprising RLuc8.6-535SG (RLuc86SG) and ALuc16. The usefulness of this BL imaging system was further shown using a multi-probe system consisting of two single-chain molecular strain probes embedding RLuc8 and ALuc23. 

This study thus proposes a unique methodology for discriminating each marine luciferase in a multi-reporter system, using the unique BL spectral signatures and intensity patterns of reporter luciferases.

## 2. Experimental Method

### 2.1. Reagents and Instrumentation for the Synthesis of the C-Series CTZ Analogues

The starting materials, reagents, and solvents were purchased from Tokyo Chemical Industry Co., Ltd., FUJIFILM Wako Pure Chemical Corporation, Kanto Chemical Co., Inc., and Sigma-Aldrich, and were used without further purification. Silica gel 70 F254 TLC plates (Wako) were used for analytical thin-layer chromatography (TLC), whereas Silica gel 60 N (spherical, neutral; Kanto Chemical) was applied in column chromatography. For preparative flash chromatography, an automated system (Smart Flash EPCLC AI-580S; Yamazen Corp., Japan) equipped with universal columns of silica gel was used. The ^1^H and ^13^C NMR spectra were determined using a JEOL ECA-500 instrument (500 MHz for 1 H and 126 MHz for 13C). Mass spectra were obtained with a high-resolution electrospray ionization mass spectrometer (JMS-T100LC; JEOL) and a matrix-assisted laser desorption/ionization mass spectrometer (JMS-S3000 SpiralTOF^TM^-plus 2.0; JEOL). 

### 2.2. Preparation of Mammalian Cell Expression Vectors Encoding Each Marine Luciferase or Single-Chain Probe

The following plasmids used in this study were obtained from our previous studies: pcDNA3.1(+) vector (Invitrogen) encoding RLuc8, RLuc86SG, NLuc, ALuc16, ALuc23, or ALuc49 [12] and pcDNA3.1(+) vector encoding each molecular tension probe, FRB-RLuc8-FKBP (F-R8-F) [20] or FRB-ALuc23-FKBP (F-A23-F) [21], whose single-chain probes were designed to sandwich a full-length marine luciferase between FRB and FKBP. After expression, rapamycin initiates an interaction between FRB and FKBP, which adds strain to the sandwiched luciferase and enhances the BL intensity.

### 2.3. Synthesis of C-Series CTZ Analogues

The C-series CTZ analogues C1–C9 were synthesized according to the following scheme (Figure S1 and Scheme S1). 2-Amino-3-benzyl-5-bromoaminopyrazine 2 was synthesized by a Negishi cross-coupling reaction of commercially available 2-amino-3,5-dibromoaminopyrazine 1 with benzyl magnesium chloride, zinc chloride, and bis (triphenylphosphine) palladium (II) dichloride. Furthermore, a Suzuki–Miyaura cross-coupling reaction was carried out using compound 2 and commercially available boronic acids B1–B8 to produce compounds 3–10. Compound 11 was prepared through a Suzuki–Miyaura cross-coupling reaction of 2-amino-3,5-dibromoaminopyrazine 1 and phenylboronic acid B9, whereas compound 12 was obtained by a Suzuki–Miyaura cross-coupling reaction with compound 11 and 4-hydroxyphenylboronic acid B10 again. Finally, compounds 3–10, 12 with ketoacetal derivatives 15 were condensed and cyclized under hydrochloric acid conditions. The synthesized CTZ analogues were named C1–C9. This term “C-series” was named after the carbon (C) elbow at the C-8 position of the imidazopyrazinone backbone.

### 2.4. Modeling of the Binding Modes of Substrates in ALuc16 and RLuc8

**Figure 1:**
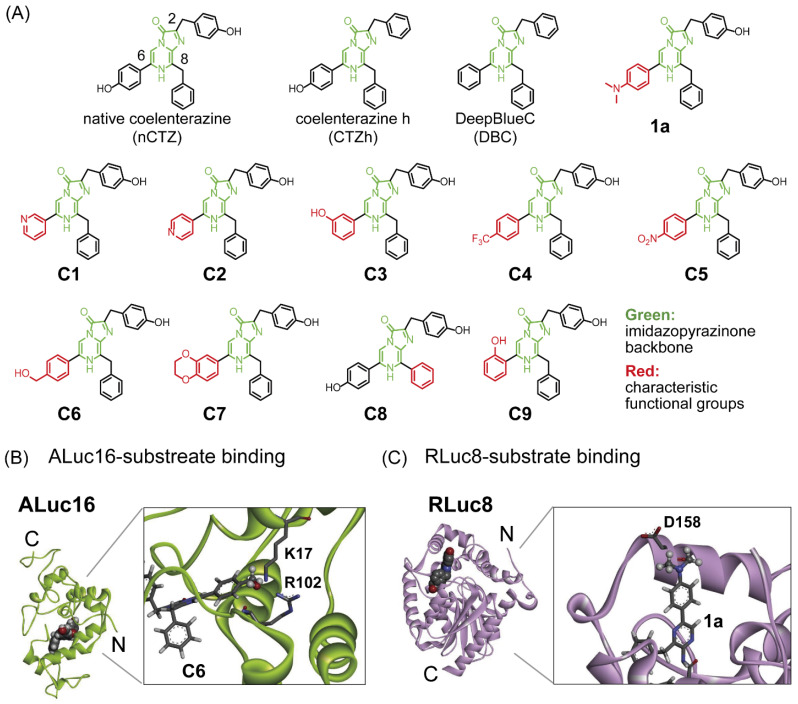
(**A**) Chemical structures of the CTZ analogues used in this study. The imidazopyrazinone backbone is highlighted in green. The characteristic functional groups are highlighted in red. Abbreviations: nCTZ, native coelenterazine; CTZh, coelenterazine h; DBC, DeepBlueC. **1a**. Our previously reported CTZ analogue, which shows the BL intensity for the RLuc series. (**B**) Three-dimensional (3D) structure of ALuc16 with nCTZ, which was modeled based on the structure of GLuc. The active site is highlighted in the box to show the specific interactions with the substrate **C6**. The hydroxymethyl (OH-CH_2_-) group of **C6** (represented by balls) was manually created by extending the corresponding functional group from the nCTZ. (**C**) The 3D structure of *Renilla* luciferase 8 (RLuc8) with coelenteramide. The active site is highlighted in the box to show the specific interactions with **1a**. The dimethylamino group ((CH_3_)_2_N-) group of **1a** (represented by balls) was manually fabricated by extending the corresponding functional group from the bound coelenteramide. Putative interacting residues are represented by sticks.

The structures of ALuc16 were created by SWISS-MODEL [22] using the model 17 of the GLuc NMR structure (PDB: 7D2O) [23] as a template (Figure 1B). The putative binding pose of nCTZ (represented by spheres) was modeled by Discovery Studio 2017 R2 (Dassault Systèmes, Vélizy-Villacoublay, France), according to the previous study [24]. Then, putative interactions of ALuc16 with C6 (extended from nCTZ) are enlarged in the box.

The structure of RLuc8 was obtained from the protein data bank (PDB: 7OMR) [25] (Figure 1C). The bound coelenteramide is presented by spheres. Then, putative interactions of RLuc8 with 1a (extended from coelenteramide) are highlighted in the box.

### 2.5. Absolute BL Intensities of the CTZ Analogues C1–C9 According to Marine Luciferases

**Figure 2:**
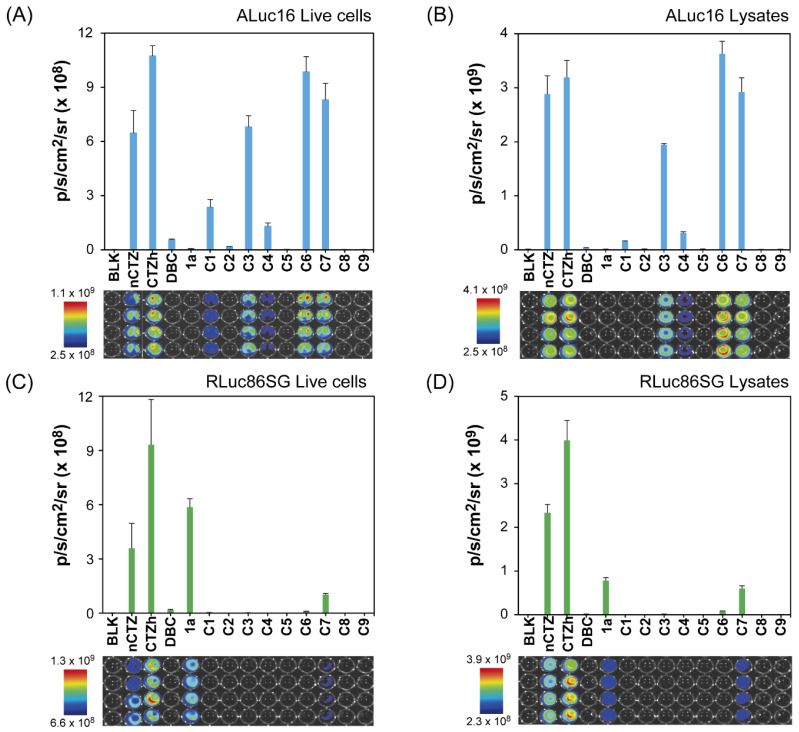
Characterization of CTZ analogues **C1**–**C9**. (**A**) The absolute BL intensities of ALuc16 in lysates according to CTZ analogues. (**B**) The absolute BL intensities of RLuc8.6SG in lysates according to CTZ analogues. (**C**) The absolute BL intensities of ALuc16 in live cells according to CTZ analogues. (**D**) The absolute BL intensities of RLuc8.6SG in live cells according to CTZ analogues. The bottom panels show the corresponding BL images.

The absolute BL intensities of the C-series CTZ analogues were determined in African green monkey kidney-derived COS-7 cells containing RLuc8.6SG or ALuc16 (Figure 2).

COS-7 cells were grown in 6-well microplates. The cells were transiently transfected with a mammalian expression vector, pcDNA3.1(+) encoding RLuc86SG or ALuc16, and incubated overnight in a humidified 5% (v/v) CO_2_ incubator. The cells were harvested by trypsinization and centrifuge, and were counted using an automatic cell counter (Countess II; Thermo Fisher Scientific). The 10^4^ number of the cells were then seeded into each well of the 96-well black-frame microplates. The cells were further incubated overnight in the CO_2_ incubator. 

The wells were conceptionally divided into two sections, and culture media were completely eliminated by suction. The cells in the wells of the first section were passively lysed with a lysis buffer (Promega) by immersing the cells in 40 μL of lysis buffer per well. The live cells in the wells of the second section were left. Both sections were then sealed before BL measurement. 

The wells of cell lysates or live cells were simultaneously injected with 40 μL of the phosphate-buffered saline (PBS) solution (hereafter referred to as substrate solution), dissolving one of the following substrates: nCTZ, coelenterazine h (CTZh), DeepBlueC (DBC), 1a, C1, C2, C3, C4, C5, C6, C7, C8, and C9, by using a 12-channel micropipette. The applied concentrations of the substrate solutions were set to 1 × 10^4^ M. The microplates were immediately set in the chamber of an IVIS imaging system (PerkinElmer). The BL images were then integrated during the initial first second, and analyzed with the specific software Living Image version 4.7.

### 2.6. Characterization of the BL Spectra of the CTZ Analogues C1–C9

**Figure 3:**
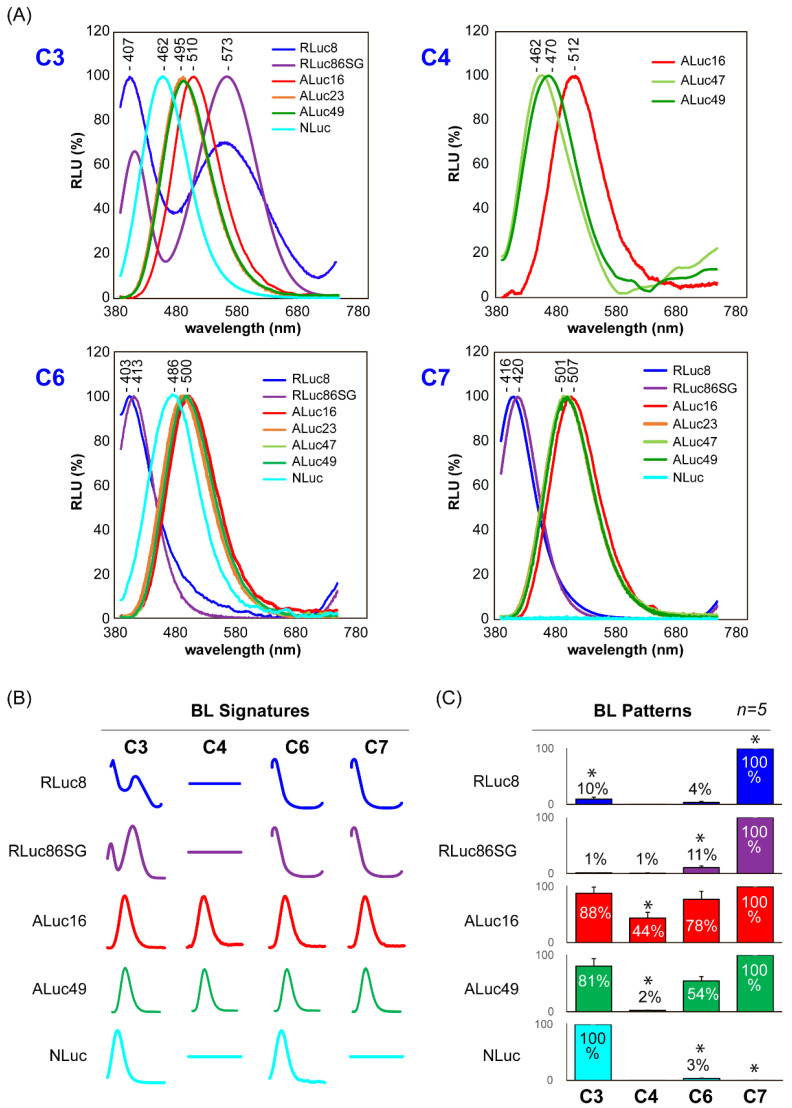
(**A**) The normalized BL spectra of various marine luciferases in the presence of the CTZ analogues C3, C4, C6, or C7. The overall BL spectra were normalized to the peak height. (**B**) BL spectral signatures of various marine luciferases in the presence of C3, C4, C6, or C7. (**C**) The patterned BL intensities of each luciferase in response to C3, C4, C6, and C7.

The BL spectra of selected C-series CTZ analogues were characterized (Figure 3 and Table 1).

COS-7 cells were cultured in 6-well microplates. When the cells reached 70% confluency, they were transiently transfected with pcDNA3.1(+) vector encoding ALuc16, ALuc23, ALuc49, RLuc8, RLuc86SG, or NanoLuc (NLuc) and then incubated for 24 h. Next, the cell culture media were completely eliminated by suction, and the remaining cells were lysed with a lysis buffer (Promega). Forty μL of the cell lysates were then aliquoted into 200 μL PCR tubes. Each tube was injected with 40 μL of the substrate solution dissolving C3, C4, C6, or C7 (final concentration: 0.5 × 10^4^ M.). The corresponding BL spectra were immediately determined with a high-precision spectrophotometer (AB-1850; ATTO) simultaneously recoding all the wavelength light at once. The total intensities of the spectra according to the substrates are compared in the pattern (Figure 3C). Table 1 shows a summary of the wavelengths of maximal intensity (λ_max_) of the spectra. 

### 2.7. Characterization of the Chemical Stability of the CTZ Analogues C3, C4, C6, and C7

The autoluminescence of the selected CTZ analogues—CTZh, C3, C4, C6, and C7—in fetal bovine serum (FBS) samples were determined as a model of the chemical stability in the physiologic samples (Figure S2 and Table S1).

FBS was diluted with PBS to make 50%, 20%, 10%, and 5% dilutions in volume percentage. Forty μL of each dilution was aliquoted to the wells of a 96-well black-frame microplate. The dilutions in wells were simultaneously injected with 40 μL of the substrate solutions containing CTZh, C3, C4, C6, C7, or a vehicle solution (PBS) (final concentration: 0.5 × 10^4^ M) using a 12-channel micropipette; the final concentrations of FBS were 25%, 10%, 5%, and 2.5% in volume percentage. The corresponding BL images of autoluminescence were determined with the IVIS imaging system and analyzed with the specific software, Living Image version 4.7.

### 2.8. Quantitative Relationship between Multi-Reporter Systems in Live Cells

**Figure 4:**
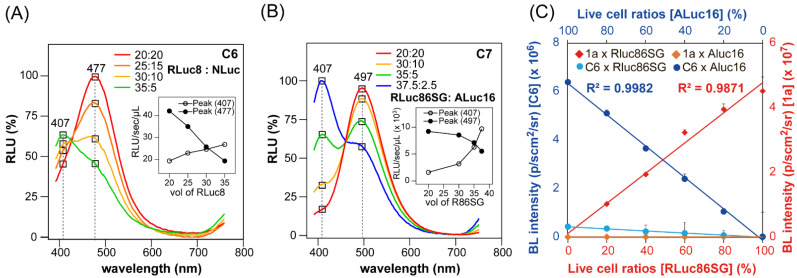
(**A**) Multi-reporter BL spectra of rational mixtures of ALuc16 and RLuc8.6SG in live COS-7 cells in the presence of **C6**. The legends denote the volume ratios of the mixtures. (**B**) Multi-reporter BL spectra of rational mixtures of ALuc16 and RLuc8.6SG in live COS-7 cells in the presence of **C7**. The peaks at 412 nm and 496 nm are the signatures of RLuc8.6SG and ALuc16, respectively. The legends denote the volume ratios of the mixtures. **C7** was chosen because it is luminescent with both ALuc16 and RLuc8.6SG, and thus is suitable for multi-reporter imaging. (**C**) Quantitative profile of the BL intensities of different ratios of live cells of ALuc16 and RLuc86SG totaling 100%. The BL intensities were developed with the substrate solutions of **1a** and **C6**. The substrates **1a** and **C6** were chosen because they are specifically bright with RLuc86SG and ALuc16, respectively.

The optical properties of multi-reporter systems were quantitatively characterized (Figure 4). The COS-7 cells containing RLuc8 or NLuc were prepared according to the protocol shown in Figure 3; they were then harvested by trypsinization and centrifuge. Next, the cells were counted with an automatic cell counter and diluted with PBS to 2.0 × 10^6^ cell/mL for RLuc8 and 2.0 × 10^6^ cell/mL for NLuc. Aliquots of the two live cells containing RLuc8 and RLuc86SG were then mixed in PCR tubes with the following ratios (in μL): 20:20, 25:15, 30:10, and 35:5, respectively. The mixtures were injected with 40 μL of the substrate solution of C6 (final concentration: 0.5 × 10^4^ M), and the BL intensities were immediately determined using a high-precision spectrophotometer. The resulting spectra were normalized to the peak intensity (Figure 4A), and the peak heights were plotted showing the mixing ratios (Figure 4A, inset).

The COS-7 cells containing ALuc16 or RLuc86SG were similarly prepared according to the protocol in Figure 3, and then harvested according to the protocol in Figure 4A. Aliquots of the two live cells containing ALuc16 and RLuc86SG were then mixed in PCR tubes with the following ratios (in μL): 20:20, 10:30, 5:35, and 2.5:37.5, respectively. The mixtures were injected with 40 μL of the substrate solution of C7 (final concentration: 0.5 × 10^4^ M), and the BL spectra were determined and analyzed according to the protocol in Figure 4A. The results are summarized in Figure 4B.

The live COS-7 cells containing ALuc16 and Rluc86SG from Figure 4B were separately diluted with PBS to 1 × 10^6^ cell/mL and 4 × 10^6^ cell/mL, respectively. The cells of ALuc16 and RLuc86SG were further diluted with PBS at different ratios totaling 100%. Ten μL of each dilution was then deployed in the wells of a 96-well black-frame microplate. The corresponding BL intensities were determined with a microplate reader (Berthold) immediately after the automatic injection of 40 μL of substrate solutions dissolving 1a or C6 (final concentration: 0.5 × 10^4^ M), followed by an analysis with the use of Excel 365 (Microsoft) (Figure 4C). 

### 2.9. Multi-Probe BL Imaging of F-R8-F and F-A23-F in Live Cells

**Figure 5:**
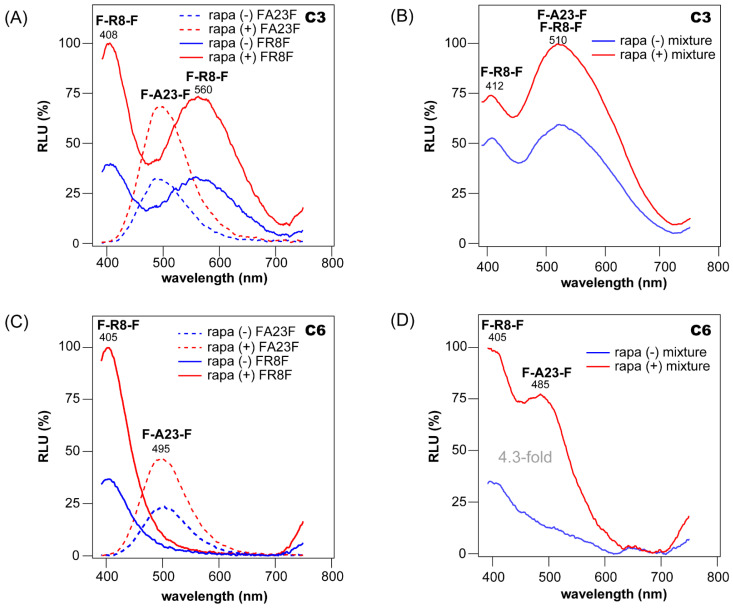
(**A**) Respective BL spectra of F-A23-F (dotted line) and F-R8-F (solid line) in the presence (+) or absence (-) of rapamycin (rapa). The BL spectra were developed with **C3**. The inset shows the working mechanism of the molecular strain probe F-R8-F. (**B**) The signatural spectra of the mixture of F-A23-F and F-R8-F in the presence or absence of rapamycin. (**C**) Respective BL spectra of F-A23-F (dotted line) and F-R8-F (solid line) in the presence (+) or absence (-) of rapamycin (rapa), which were illuminated by **C6**. The inset shows the working mechanism of the molecular strain probe F-A23-F. (**D**) The signatural spectra of the mixture of F-A23-F and F-R8-F in the presence or absence of rapamycin.

Multi-probe BL imaging was carried out on the mixtures of COS-7 cells expressing F-R8-F and F-A23-F (Figure 5). 

The COS-7 cells were cultured in a 6-well microplate and transiently transfected with pcDNA3.1(+) vector encoding F-R8-F or F-A23-F; they were then incubated overnight in a CO_2_ incubator. Next, the cells were stimulated with 10^−7^ M rapamycin or its vehicle (0.1% ethanol as final concentration) and again incubated overnight. The cells with and without rapamycin were harvested by trypsinization and centrifuge. The rapamycin-stimulated or rapamycin-free cells expressing F-R8-F or F-A23-F were resuspended with PBS to 7 × 10^6^ cells. Forty μL of each resuspension was aliquoted into a PCR tube. Separately, 40 μL of the rapamycin-stimulated cells expressing F-R8-F were mixed in a PCR tube, with 20 μL of the rapamycin-stimulated cells containing F-A23-F. The same ratio of the mixture was prepared in a PCR tube using the rapamycin-free cells containing F-R8-F and F-A23-F. 

Each PCR tube prepared above was injected with 40 μL of the substrate solution of C3 or C6 (final concentration: 0.5 × 10^4^ M). The corresponding BL spectra were immediately determined with the precision spectrophotometer.

## 3. Results and Discussion

### 3.1. Molecular Designs of the Novel C-Series CTZ Analogues (C1–C9)

In the present study, a series of CTZ analogues (named C1–C7 and C9) were synthesized by modifying the functional group at the C-6 position of the nCTZ backbone (Figure 1). For the substrate design, we referred to a previous study on the X-ray crystallographic information of RLuc [26], in which the active site of RLuc accommodates the specific substrate nCTZ from the C-6 position. Therefore, the substrates were created by modifying the C-6 position with the expectation of specificity to RLuc and/or ALucs. In the molecular design, a bulky functional group such as the benzodioxans was introduced at the C-6 position. On the other hand, the CTZ analogue C8 was synthesized by substituting the benzyl structure at the C-8 position with a phenyl structure (Figure 1). This synthesis was carried out with the expectation of the spectral redshift and specificity to a specific luciferase. The synthesized substrates (C1–C9) were named “C-series.”

### 3.2. Characterization of the C-Series CTZ Analogues C1–C9

The BL properties of the novel CTZ analogues C1–C9, together with conventional nCTZ, CTZh, DBC, and 1a, were characterized with mammalian COS-7 cells containing ALuc16, RLuc86SG, or NLuc (Figure 2). In this characterization, the CTZ analogue 1a was chosen because it strongly luminesced with RLuc variants in our previous study [10] (putative interactions with 1a are modeled in Figure 1C). 

The quantitative analysis showed that the analogues C3, C6, and C7 were exceptionally bright with ALuc16 compared with RLuc86SG, in both live cells and lysates: e.g., C6 and C7 luminesced as strongly as nCTZ with ALuc16 (putative interactions with C6 are modeled in Figure 1B). C3 and 1a were specific to ALuc16 and RLuc86SG, respectively. 

Interestingly, the only difference among nCTZ, C3, and C9 is that their hydroxyl (OH) group is in the *p*-, *m-*, and *o*-positions, respectively. Nevertheless, this small structural variance resulted in considerable variance in selectivity to marine luciferases: i.e., nCTZ was bright with both ALuc16 and RLuc86SG, C3 was specifically bright with ALuc16, and C9 was completely dark with both ALuc16 and RLuc86SG. These features were observed in both live cells and lysates.

C1 and C4 developed weak but ALuc16-specific BL intensity in both live cells and lysates. However, most of the C-series analogues did not show significant BL intensities with RLuc86SG, except C7, which luminesced with approximately 25% of the BL intensity of nCTZ. It is unclear why C7 showed such a distinctive activity feature with RLuc86SG, which was different from the others. In the literature, it has previously been reported that CTZ analogues carrying an ether group at the C-6 position were generally bright with RLuc analogues [27]. C7 also has a circular ether group at the C-6 position, and was found to be reactive with RLuc86SG. Additional corresponding studies may be helpful for decoding the detailed quantitative structure activity relationship (QSAR).

In parallel, the analogues C1–C9 were applied to NLuc, and C3 and C6 were found to exert weak absolute BL intensities (Figure S3).

As shown in Figure 2, C1, C3, C4, and C6 are specific to ALuc16. This ALuc16 specificity may be explained by the molecular design of the CTZ analogues. In the X-ray crystallographic information of RLuc, the C-6 position of nCTZ was identified as a deep site of the active site pocket of RLuc [26]. Considering that they comprise a bulky functional group at the C-6 position, C-series analogues are prone to the influence of steric hindrance on binding RLuc8. On the other hand, such a functional group at the C-6 position of CTZ is simulated to be located at the outer site of the active site pocket when it binds ALuc [26]. This binding modality supports the distinctive luciferase specificity of **C6** and **C7** to ALuc16 and RLuc86SG.

### 3.3. Characterization of the Autoluminescence Properties of the C-Series CTZ Analogues

nCTZ and its analogues are known to decompose in serum, as has been observed with autoluminescence. Regarding bioavailability, it is important to check if the prepared CTZ analogues exert autoluminescence in serum. The autoluminescence of the selected C-series CTZ analogues C3, C4, C6, and C7 was characterized by using fetal bovine serum (FBS) (Supplementary Figure S2 and Supplementary Table S1).

Among the four C-series CTZ analogues, the strongest autoluminescence was observed with C7, and the intensities were proportional to the applied FBS volumes. Nevertheless, the autoluminescence intensity was merely 37% of that of CTZh. The other C-series CTZ analogues, C3, C4, and C6, did not emit notable autoluminescence with FBS: e.g., C4 emitted merely 0.05% of the autoluminescence signal of CTZh.

The autoluminescence mechanism of C7 can be explained by the serum albumin; that is, when albumin takes a major portion of the serum proteins, a lipophobic site is exposed, which contributes to the binding and decomposition of CTZs. In particular, C7 has a benzodioxan structure at the C-6 position, which is a functional group known to be sensitive to serum albumin [28].

### 3.4. BL Spectral Signatures of the Selected CTZ Analogues C3, C4, C6, and C7

The BL spectral properties of four C-series CTZ analogues: C3, C4, C6, and C7, were investigated in the presence of various marine luciferases (Figure 3). 

The most unique signatural spectra according to marine luciferases were observed with C3. RLuc8 specifically emitted three signatural peaks at 407, 573, and 790 nm with C3; the peak at 407 nm was the highest, whereas the peak at 790 nm was considered a bouncing peak (instrumental noise) of that at 407 nm. In addition, RLuc86SG luminesced with two signatural peaks at 407 and 573 nm with C3, with the peak at 573 nm being the strongest. 

In contrast to the RLuc-series luciferases, the copepod-derived luciferases ALuc23 and ALuc16 showed only a single signatural peak with C3 at 495 and 510 nm, respectively. NLuc also generated a single signatural peak at 462 nm with C3. These singular peaks discriminate each luciferase with the specific peak positions. 

Collectively, the results indicate that the applied marine luciferases are distinguishable from each other through their spectral characteristics, including their peak positions and intensity profiles (named “spectral signatures”). 

ALuc16 showed a significant single spectral peak at 512 nm with C4. On the other hand, ALuc47 and ALuc49 generated a weak single spectral peak at 462 and 470 nm, respectively, with absolute intensities that were less than 20% of that of ALuc16. The peak wavelengths were also approximately 50 nm shorter than that of ALuc16. 

The marine luciferases showed completely different spectral signatures with C6 and C7 compared with C3. Both RLuc8 and RLuc86SG generated two signatural peaks, with the major peaks found at 403 and 413 nm, respectively. The minor peaks observed at 790 nm appeared to be the instrumental noise of the major peak at 403 and 413 nm. The RLuc variants did not generate any signatural peak at around 560 nm with C6 and C7, as originally observed with C3. 

In contrast, the other marine luciferases, NLuc and ALucs, luminesced with a single, bluish-green peak with C6 and C7. NLuc exerted a signatural BL peak at 486 nm with C6. The peaks with C6 and C7 were 24 and 39 nm redshifted peaks, respectively, compared with the peak observed with C3. The ALuc-series luciferases did not show a significant spectral shift with C6 and C7 compared with the peak with C3. The peaks of the ALuc spectra were all singular and located near 500 nm. Table 1 shows the peak positions according to the substrates: C3, C4, C6, and C7.

These results led to the idea that the spectral peak positions and intensity profiles could be used as unique signatures to distinguish each luciferase from the others. This basic idea is summarized in the simplified signatures in Figure 3B. 

Furthermore, the total intensities of the BL spectra according to the substrates C3, C4, C6, and C7 were found to collectively form unique patterns, as shown in Figure 3C. The patterns showed that RLucs were dominantly bright with C7, whereas NLuc luminesced with exceptional brightness only with C3. RLuc8 was distinguishable from RLuc86SG by to its pair BL intensity with C3 (10%), together with its notably poor intensity with C6. In contrast, RLuc86SG was different from RLuc8 by its pair intensity with C6 (11%) and exceptionally poor intensity with C3. ALuc16 was commonly bright with all the applied substrates, although its brightness somewhat varied according to the substrates. ALuc49 was distinguishable from ALuc16 by its notably poor intensity with C3 and highest intensity with C7.

The spectral signatures, together with the unique intensity patterns of each luciferase, enabled each luciferase to be distinguished from the others. 

The blue- or redshifted peaks may be explained by the multiple energy levels of the intermediates of the C-series CTZ analogues. nCTZ is known to show broad emission peaks from blue to green (400~535 nm) because of the four different energy levels of the intermediates, i.e., neutral species, amide anion, phenolate anion, and pyrazine anion [29]. The original emission peaks with nCTZ should appear as more blue- or redshifted with the C-series CTZ analogues. This is due to their variation in the π conjugation and the character of the functional groups, such as electron-donating or -withdrawing groups. 

This view may be applied to the explanation of the peak shifts of ALuc16, ALuc47, ALuc49, RLuc8, and RLuc86SG: ALucs did not show dramatic blue- or redshifted peaks with the C-series CTZ analogues compared with nCTZ. Their peaks near 500 nm are interpreted as ALucs generating amide anions as the major intermediate in the reaction with C-series CTZ analogues. In contrast, RLuc8 and RLuc86SG showed a dramatic variance in peak position with the C-series CTZ analogues. Their blue-shifted peaks near 410 nm are attributed to the fact that they generate only neutral species as the major intermediate with **C6** and **C7**. This is because **C6** and **C7** have no direct OH group at the C-6 position, and thus mostly create neutral forms with RLucs, which is known to have an emission peak at around 386–423 nm. Interestingly, RLuc8 and RLuc86SG showed two major peaks at 407 and 573 nm with **C3**. These results are thought to be caused by the generation of both neutral and phenolate/pyrazine anion forms with **C3** containing an OH group at the C-6 position. This seems to be a unique property of **C3**, different from the other C-series CTZ analogues.

### 3.5. Quantitative Relationship between Reporters in Multi-Reporter Systems

The quantitative relationship among reporter luciferases was determined with the mixtures of COS-7 cells containing various marine luciferases (Figure 4).

As the first multi-reporter system, a series of the mixtures of COS-7 cells containing RLuc8 and NLuc were luminesced with C6 (Figure 4A). The results show that the 20:20 mixture developed a unique peak at 477 nm, whereas the 35:5 mixture specified another unique peak at 407 nm. The spectral peaks at 477 and 405 nm represented the spectra of NLuc and RLuc8 developed with C6. For the other mixtures, the spectral peaks were observed between the peaks of the 20:20 and 35:5 mixtures. The peak heights at 477 and 407 nm showed linearity according to the applied volumes of the cells containing luciferases (Figure 4A, inset). The two linear lines formed a cross near the point of the 30:10 mixture. The peak gap was found at around 70 nm.

As the second multi-reporter system, the mixtures of COS-7 cells containing RLuc86SG and ALuc16 were luminesced with C7 (Figure 4A). The results show that the 20:20 mixture developed a unique peak at 497, which gradually decreased with the decreased amount of ALuc16 cells in the mixture. Due to the amount of RLuc86SG cells in the ratio, the peak at 407 nm was observed to be dominant. The highest peak at 407 nm was found with the 37.5:2.5 mixture. Figure 4B (inset) presents a summary of the overall peak heights at 497 and 407 nm. The peak heights showed a linear–curve relationship with the applied volumes of the cells containing luciferases. The curve lines formed a cross near the point of the 35:5 mixture. The peak gap was found at around 90 nm.

Collectively, the results in Figure 4A,B confirm that (i) the CTZ analogues C6 and C7 are useful in discriminating each peak in multi-reporter systems, and (ii) the separated peaks and the spectral dynamics of C7 indicate that C7 is an optimal substrate for multi-reporter systems using RLuc- and ALuc-series reporters. 

The quantitative relationship between ALuc16 and RLuc86SG in COS-7 cells was investigated in the presence of C6 and 1a; the substrates C6 and 1a were chosen because they were specifically bright with ALuc16 and RLuc86SG, respectively (Figure 4C). The results show that the substrate 1a selectively developed BL intensities with RLuc86SG but was dark with ALuc16. On the other hand, C6 preferably luminesced with ALuc16, but less so with RLuc86SG. The 1a–RLuc86SG combination was approximately 7-fold brighter than the **C6**–ALuc16 combination, as shown in the Y-axis scales. 

### 3.6. Multi-Probe BL Imaging with COS-7 Cells Containing F-R8-F and F-A23-F

A multi-probe BL imaging system was developed with COS-7 cells containing F-R8-F and F-A23-F (Figure 5).

First, an examination was carried out to determine if each single-chain probe, F-R8-F or F-A23-F, worked properly in response to rapamycin (Figure 5A,C). The results show that F-R8-F in live cells developed two signatural peaks at 408 and 560 nm, with C3 or one signatural peak at 405 nm with C6. The overall spectral intensities were enhanced more than 2-fold after stimulation with rapamycin. Meanwhile, F-A23-F exerted a signatural peak at 500 nm with nCTZ, the position of which was not significantly influenced by replacing the substrate C3 with C6. 

Second, the BL spectra of the mixture of cells carrying F-R8-F and F-A23-F in the presence of C3 and C6 were determined (Figure 5B,D).

The results show that, in the presence of C3, the mixture had two signatural peaks at 412 and 510 nm; the peak at 412 nm originated from F-R8-F, but the peak at 510 nm was unclear. The unclear peak at 510 nm is interpreted as a combined peak of F-A23-F (495 nm) and F-R8-F (560 nm). On the other hand, the mixture developed two signatural peaks at 405 and 485 nm in the presence of C6. This is interpreted as an indication that the peak at 405 nm is from F-R8-F, whereas the peak at 485 nm is from F-A23-F. The peak position of F-A23-F in the multiplex system was slightly blue-shifted (485 nm) compared with the peak of F-A23-F alone. This apparent blueshift is considered by a doubling effect of the two superimposed spectra. The mixture enhanced the BL intensities up to 4.3-fold after the addition of rapamycin. 

The results for C3 and C6 indicate that (i) the multi-probe system generates unique BL spectra with signatural peaks at defined positions, (ii) the multi-probe system recognizes its specific ligand and dramatically enhances the BL intensities, and (iii) nCTZ cannot discriminate the spectral peaks of F-A23-F and F-R8-F because they are spectrally too close each other (Table 1). However, in the case of C3 and C6, the spectral peaks are separately detectable with optical filters, or collectively identified by their distinctive signatural spectra in pattern.

## 4. Conclusions

In this study, a unique BL signature imaging system with novel CTZ analogues named “C-series” was developed. The BL spectra and intensity patterns of nine kinds of the C-series CTZ analogues were first characterized according to the luciferases. The selected CTZ analogues, C3, C4, C6, and C7, were found to individually or collectively create unique BL spectral signatures and intensity patterns according to each marine luciferase. The quantitative properties of the spectral signatures and intensity patterns were characterized with individual or multiple reporter luciferases. The usefulness of the system was shown with the use of two single-chain molecular strain probes embedding RLuc8 and ALuc23. This study provides unique methodological insights on how to construct a BL signature imaging system for specifying marine luciferases by using their unique BL signatures and intensity patterns. The usefulness of this BL signature imaging system may be expanded to an animal modality, to decode specific reporter signals in the complex context of a biological system.

## Figures and Tables

**Table 1:** The maximal BL wavelengths (λ_max_) of various marine luciferases in the presence of the CTZ analogues **C3**, **C4**, **C6**, and **C7**.

Luciferase	Substrates (nm)				
	nCTZ	C3	C4	C6	C7
ALuc16	496	510	512	500	507
ALuc23	500	495	-	496	499
F-A23-F	500	499	500	498	499
ALuc47	490	490	462	502	501
ALuc49	490	495	470	500	501
RLuc8	480	407, 567	-	403	416
F-R8-F	490	407, 564	-	403	412
RLuc8.6SG	535	408, 573	415	413	420
NanoLuc		462	-	465	-

## Supplementary Materials

The following supporting information can be downloaded at: https://www.mdpi.com/article/10.3390/ijms232113047/s1.
