# Supplementary material for: C-Series Coelenterazine-Driven Bioluminescence Signature Imaging
Source: Int J Mol Sci. 2022 Oct 27;23(21):13047. doi: 10.3390/ijms232113047 (PMC9658928; doi:10.3390/ijms232113047)
Supplement: Supplementary file 1 [file ijms-23-13047-s001.zip › ijms-1980008-supplementary.pdf]

International Journal of *Molecular Sciences*

Supplementary

C-Series Coelenterazine-Driven Bioluminescence Signature Imaging

Genta Kamiya [1], Nobuo Kitada [1], Tadaomi Furuta [2], Takashi Hirano[1], Shojiro Maki [1] and Sung Bae Kim [3,*]

[1] Department of Engineering Science, Graduate School of Informatics and Engineering, The University of Electro-Communications, Chofu, Tokyo, 182-8585, Japan
[2.] School of Life Science and Technology, Tokyo Institute of Technology, B-62 4259 Nagatsuta-cho, Midori-ku, Yokohama 226-8501, Japan
[3.] Research Institute for Environmental Management Technology, National Institute of Advanced Industrial Science and Technology (AIST), 16-1 Onogawa, Tsukuba 305-8569, Japan
* Corresponding author: kimu-sb@aist.go.jp.

Contents

1. Brief illustration of the synthetic scheme of the C-series analogues (Fig. S1) page 2
2. Autoluminescence intensities of CTZ analogues **C3**, **C4**, **C6**, and **C7** according to the contents of fetal bovine serum (FBS) (Fig. S2) page 3
3. Table of the autoluminescence intensities of the selected CTZ analogues according to the contents of FBS (Table S1) page 4
4. Absolute BL intensities of CTZ analogues **C1–C9** with NanoLuc (Fig. S3) page 5
5. The specific procedure of the organic synthesis and the corresponding NMR data of the C-series CTZ analogues (Scheme S1) page 6

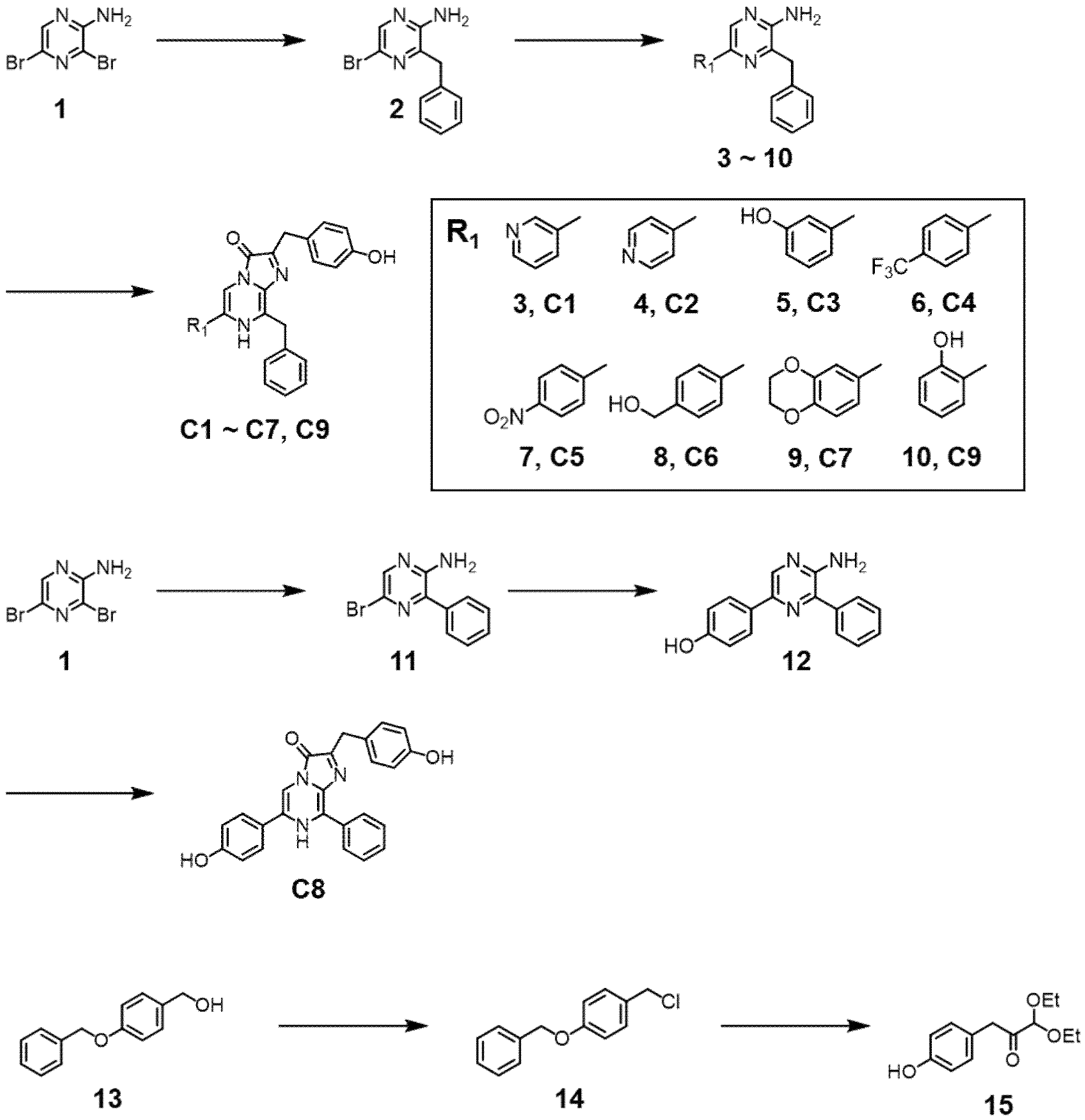

Figure S1. Brief illustration of the synthetic scheme of the C-series analogues.

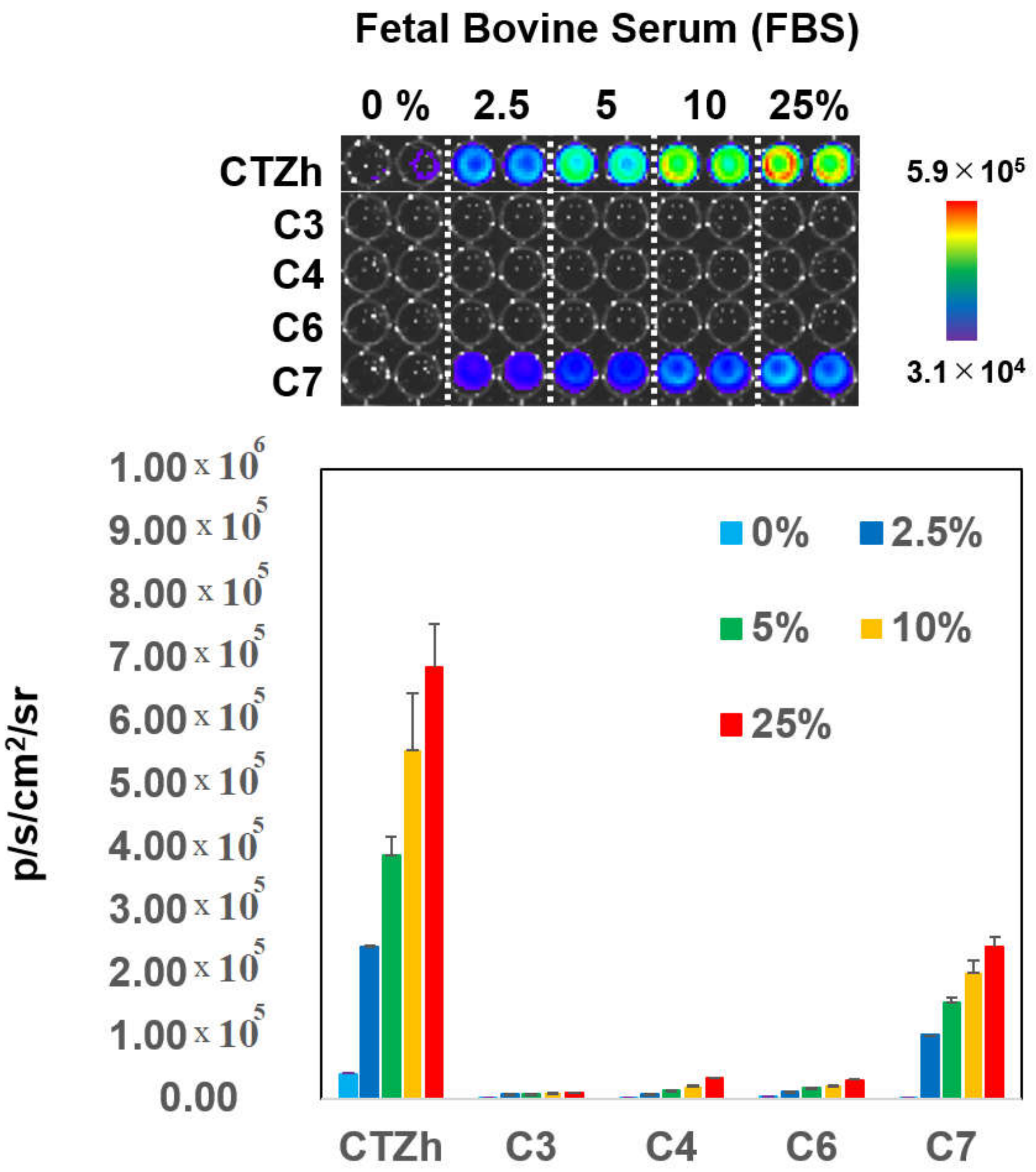

Figure S2. Autoluminescence intensities of CTZ analogues **C3**, **C4**, **C6**, and **C7** according to the contents of fetal bovine serum (FBS). The percentages in the annotation denote the final contents of FBS in the measurement solutions.

Table S1. The absolute intensities of autoluminescence according to the contents of fetal bovine serum (FBS).

**Substrate**	**Fetal bovine serum (FBS, %)**				
	**0%**	**2.5%**	**5%**	**10%**	**25%**
**CTZh**	$4.0 \pm 0.2 \times 10^4$	$24.1 \pm 0.2 \times 10^4$	$38.7 \pm 3.0 \times 10^4$	$55.4 \pm 9.0 \times 10^4$	$68.5 \pm 6.8 \times 10^4$
**C3**	$2.7 \pm 0.4 \times 10^3$	$7.5 \pm 0.0 \times 10^3$	$8.5 \pm 0.2 \times 10^3$	$9.5 \pm 0.1 \times 10^3$	$10.7 \pm 0.1 \times 10^3$
**C4**	$1.7 \pm 0.2 \times 10^3$	$8.6 \pm 0.3 \times 10^3$	$13.9 \pm 0.2 \times 10^3$	$19.6 \pm 2.0 \times 10^3$	$33.0 \pm 1.2 \times 10^3$
**C6**	$3.4 \pm 0.2 \times 10^3$	$12.5 \pm 0.1 \times 10^3$	$17.5 \pm 0.6 \times 10^3$	$21.0 \pm 1.5 \times 10^3$	$29.7 \pm 1.5 \times 10^3$
**C7**	$2.8 \pm 0.2 \times 10^3$	$1.0 \pm 0.0 \times 10^5$	$1.5 \pm 0.1 \times 10^5$	$2.0 \pm 0.2 \times 10^5$	$2.4 \pm 0.2 \times 10^5$
Unit: p/sec/cm^2/sr					

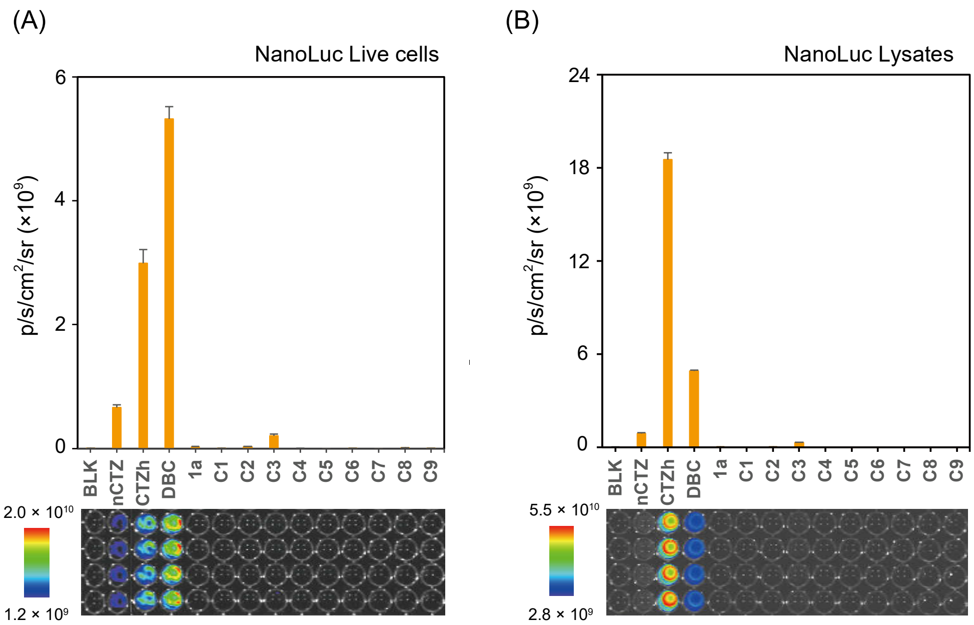

Figure S3. Absolute BL intensities of CTZ analogues **C1–C9** with NanoLuc. The absolute intensities were specified in average radiance values (p/s/cm²/sr). The bottom panel shows the corresponding BL images of each substrate.

Scheme S1. The specific procedure of the organic synthesis and the corresponding NMR data of the C-series CTZ analogues.

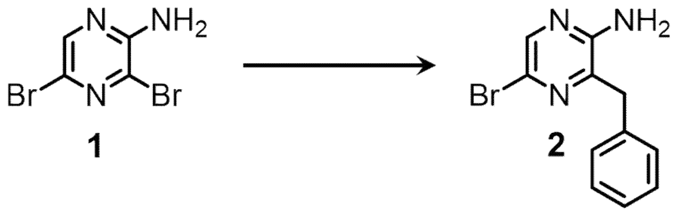

3-benzyl-5-bromopyrazin-2-amine 2

Zine chloride (2.7 g, 19.77 mmol) and 1M Benzylmagnesium chloride solution in THF (22 mL, 19.77 mmol) stirred for 1 hour at room temperature under argon. Bis(triphenylphosphine)palladium(II) Dichloride (351 mg, 0.49 mmol) and 3,5-dibromopyrazine-2-amine **1** (2.5 g, 9.89 mmol) dissolved in THF (22 mL) were added to the mixture for 4 days at room temperature under argon. After the reaction was completed, water was added, and the product was extracted with ethyl acetate (200 ml × 3). The organic phase dried over anhydrous sodium sulfate and evaporated. The residue was separated by column chromatography (hexane / ethyl acetate = 4/1 to 3/1) to obtain compound **2** (2.1 g, 7.98 mmol, 81%, yellow solid).

^{1}H-NMR (500 MHz, CHLOROFORM-D) δ 8.03 (s, 1H), 7.34-7.21 (m, 5H), 4.41 (s, 2H), 4.08 (s, 2H)
^{13}C-NMR (126 MHz, CHLOROFORM-D) δ 152.3, 142.7, 141.9, 135.9, 129.3, 128.6, 127.5, 126.5, 41.0
HR-ESI-MS: *m/z*: [M+H]+ calcd for $C_{11}H_{11}{}^{79}Br_{1}N_{3}$, 264.01363; found, 264.01253
HR-ESI-MS: *m/z*: [M+H]+ calcd for $C_{11}H_{11}{}^{81}Br_{1}N_{3}$, 266.01103; found, 266.01159

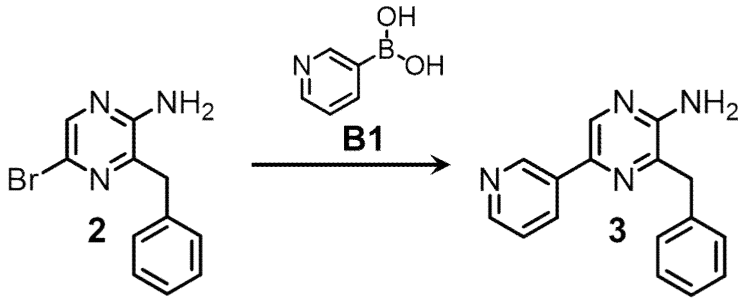

3-benzyl-5-(pyridin-4-yl)pyrazin-2-amine 3

Compound **2** (100 mg, 0.38 mmol) and 4-Pyridylboronic Acid **B1** (60 mg, 0.49 mmol) were dissolved in 1,4-dioxane (2 mL) at room temperature. Tetrakis(triphenylphosphine)palladium(0) (21 mg, 0.019 mmol) and Na_2CO_3 aq (2 mL) were added to the mixture for 2 hour at 110 °C under argon. After the reaction was completed, 30 mL of water was added, and the product was extracted with ethyl acetate (2 × 60 mL). The organic phase was dried over anhydrous sodium sulfate and evaporated. The residue was separated by column chromatography (hexane / ethyl acetate = 1/1 to ethyl acetate) to obtain compound **3** (66 mg, 0.25 mmol, 67%, yellow crystals).

^{1}H-NMR (500 MHz, CHLOROFORM-D) δ 9.17 (d, J = 1.7 Hz, 1H), 8.60 (q, J = 2.1 Hz, 1H), 8.42 (s, 1H), 8.25 (dt, J = 8.0, 2.0 Hz, 1H), 7.55 (t, J = 6.6 Hz, 1H), 7.48-7.45 (m, 2H), 7.34 (t, J = 7.4 Hz, 1H), 7.28 (d, J = 4.6 Hz, 2H), 4.51 (s, 2H), 4.19 (s, 2H)

^{13}C-NMR (126 MHz, CHLOROFORM-D) δ 152.4, 149.2, 147.3, 141.3, 139.8, 137.8, 136.5, 132.3, 132.2, 129.2, 128.7, 127.3, 123.7, 41.3

HR-ESI-MS: *m/z*: [M+H]+ calcd for $C_{16}H_{15}N_4$, 263.13021; found, 263.12967

Compounds **4-12** were prepared with a similar procedure to the preparation of **3**.

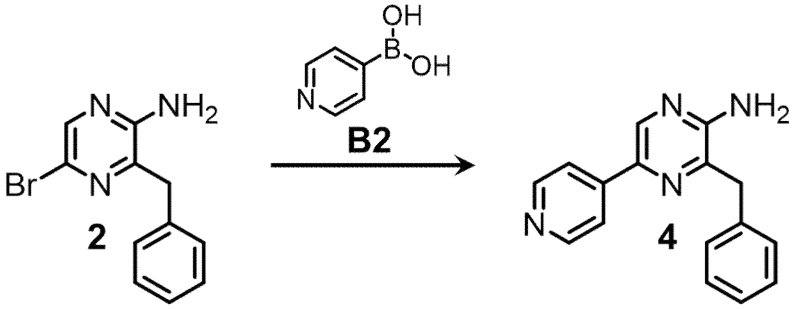

3-benzyl-5-(pyridin-3-yl)pyrazin-2-amine 4

^{1}H-NMR (500 MHz, CHLOROFORM-D) δ 8.67 (q, J = 2.1 Hz, 2H), 8.49 (s, 1H), 7.86 (q, J = 2.1 Hz, 2H), 7.36-7.27 (m, 4H), 4.60 (s, 2H), 4.20 (s, 2H)

^{13}C-NMR (126 MHz, CHLOROFORM-D) δ 153.0, 150.4, 144.4, 141.1, 139.1, 138.2, 132.1, 129.1, 128.6, 127.3, 119.7, 41.2

HR-ESI-MS: *m/z*: [M+H]+ calcd for $C_{16}H_{15}N_4$, 263.12982; found, 263.12967

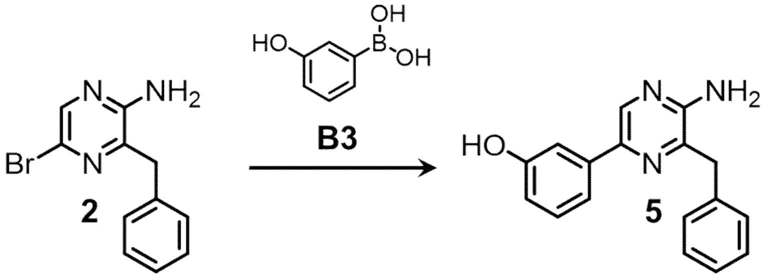

3-(5-amino-6-benzylpyrazin-2-yl)phenol 5

^{1}H-NMR (500 MHz, ACETONE-D6) δ 8.38 (s, 1H), 8.35 (s, 1H), 7.52 (t, J = 2.0 Hz, 1H), 7.45 (dd, J = 8.9, 1.4 Hz, 1H), 7.37 (d, J = 6.9 Hz, 2H), 7.31-7.28 (m, 2H), 7.26-7.20 (m, 2H), 6.81 (q, J = 3.6 Hz, 1H), 5.62 (s, 2H), 4.17 (s, 2H)

^{13}C-NMR (126 MHz, ACETONE-D6) δ 158.7, 153.6, 141.5, 140.9, 140.0, 138.9, 138.1, 130.5, 129.7, 129.3, 127.3, 117.3, 115.5, 113.1, 40.5

HR-ESI-MS: *m/z*: [M+H]+ calcd for $C_{17}H_{16}N_3O_1$, 278.12913; found, 278.12934

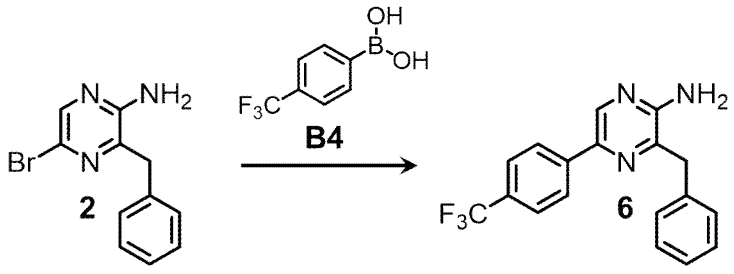

3-benzyl-5-(4-(trifluoromethyl)phenyl)pyrazin-2-amine 6

^{1}H-NMR (500 MHz, ACETONE-D6) δ 8.56 (s, 1H), 8.21 (d, J = 8.0 Hz, 2H), 7.76 (d, J = 8.0 Hz, 2H), 7.38 (d, J = 8.0 Hz, 2H), 7.31 (t, J = 7.4 Hz, 2H), 7.23 (t, J = 7.4 Hz, 1H), 5.86 (s, 2H), 5.63 (s, 1H)

^{13}C-NMR (126 MHz, CHLOROFORM-D) δ 152.3, 140.9, 140.8, 140.6, 137.9, 136.5, 129.1, 128.6, 127.2, 125.8, 125.7, 125.7, 41.2

HR-ESI-MS: *m/z*: [M+H]+ calcd for $C_{18}H_{15}F_3N_3$, 330.12290; found, 330.12181

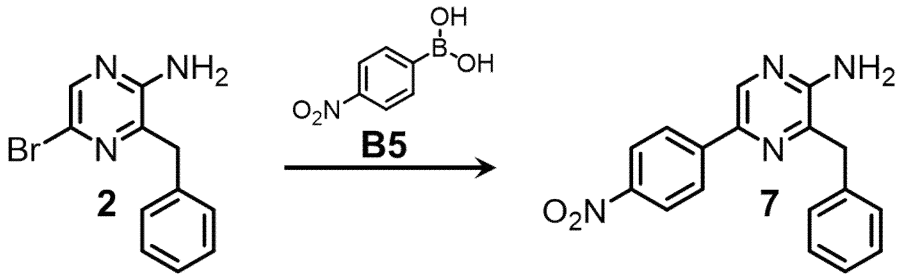

3-benzyl-5-(4-nitrophenyl)pyrazin-2-amine 7

^{1}H-NMR (500 MHz, CHLOROFORM-D) δ 8.49 (s, 1H), 8.30 (dd, J = 11.5, 2.3 Hz, 2H), 8.12 (dd, J = 11.2, 2.6 Hz, 2H), 7.36-7.27 (m, 5H), 4.61 (s, 2H), 4.20 (s, 2H)

^{13}C-NMR (126 MHz, CHLOROFORM-D) δ 152.7, 147.4, 143.4, 141.2, 139.6, 138.5, 136.2, 129.2, 128.6, 127.3, 126.0, 124.2, 41.2

HR-ESI-MS: *m/z*: [M+H]+ calcd for $C_{17}H_{15}N_4O_2$, 307.12044; found, 307.11950

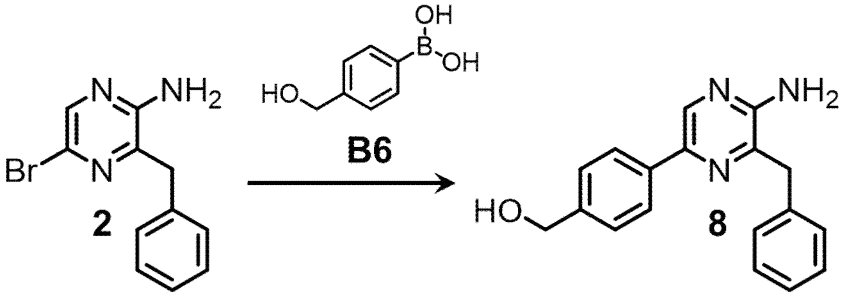

(4-(5-amino-6-benzylpyrazin-2-yl)phenyl)methanol 8

^{1}H-NMR (500 MHz, CHLOROFORM-D) δ 8.38 (s, 1H), 7.94 (d, J = 8.6 Hz, 2H), 7.46 (d, J = 8.6 Hz, 2H), 7.34-7.28 (m, 5H), 4.75 (d, J = 5.2 Hz, 2H), 4.41 (s, 2H), 4.19 (s, 2H)

^{13}C-NMR (126 MHz, ACETONE-D6) δ 153.5, 142.9, 141.6, 141.0, 138.8, 137.9, 137.1, 129.7, 129.3, 127.7, 127.2, 125.9, 64.5, 40.5

HR-ESI-MS: *m/z*: [M+H]+ calcd for $C_{18}H_{18}N_3O_1$, 292.14503; found, 292.14499

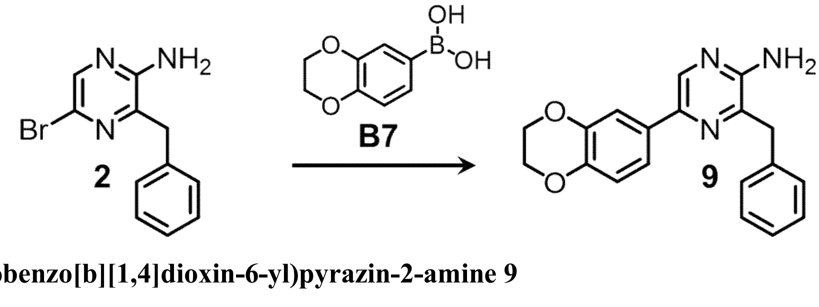

3-benzyl-5-(2,3-dihydrobenzo[b][1,4]dioxin-6-yl)pyrazin-2-amine 9

^{1}H-NMR (500 MHz, ACETONE-D6) δ 8.35 (s, 1H), 7.48 (d, J = 2.3 Hz, 1H), 7.45 (dd, J = 8.3, 2.0 Hz, 1H), 7.37 (d, J = 8.0 Hz, 2H), 7.30 (t, J = 7.7 Hz, 2H), 7.21 (t, J = 7.4 Hz, 1H), 6.87 (d, J = 8.6 Hz, 1H), 5.54 (s, 1H), 4.29 (s, 4H), 4.16 (s, 2H)
^{13}C-NMR (126 MHz, CHLOROFORM-D) δ 151.4, 143.9, 142.4, 140.6, 137.0, 136.9, 131.1, 129.1, 128.7, 127.1, 119.1, 117.7, 114.8, 64.6, 64.5, 41.3

HR-ESI-MS: *m/z*: [M+H]+ calcd for $C_{19}H_{18}N_{3}O_{2}$, 320.13956; found, 320.13990

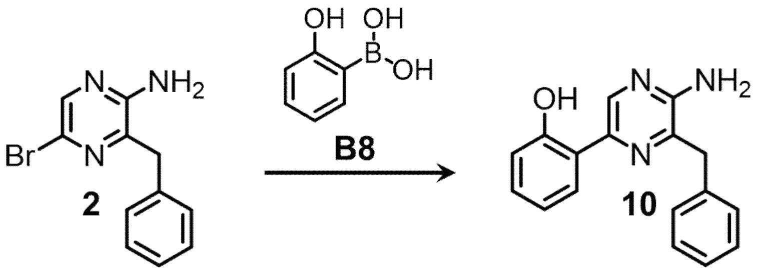

2-(5-amino-6-benzylpyrazin-2-yl)phenol 10
^{1}H-NMR (500 MHz, CHLOROFORM-D) δ 8.62 (s, 1H), 7.73 (dd, J = 8.0, 1.7 Hz, 1H), 7.37 (t, J = 7.2 Hz, 2H), 7.31 (t, J = 7.4 Hz, 1H), 7.25-7.22 (m, 3H), 6.97 (d, J = 8.0 Hz, 1H), 6.92-6.89 (m, 1H), 4.53 (s, 2H), 4.12 (s, 2H)
^{13}C-NMR (126 MHz, CHLOROFORM-D) δ 158.0, 151.0, 142.7, 137.7, 136.8, 135.7, 130.3, 129.3, 128.9, 127.5, 124.8, 119.3, 118.4, 118.1, 40.2
HR-ESI-MS: *m/z*: [M+H]+ calcd for $C_{17}H_{16}N_{3}O_{1}$, 278.12940; found, 278.12934

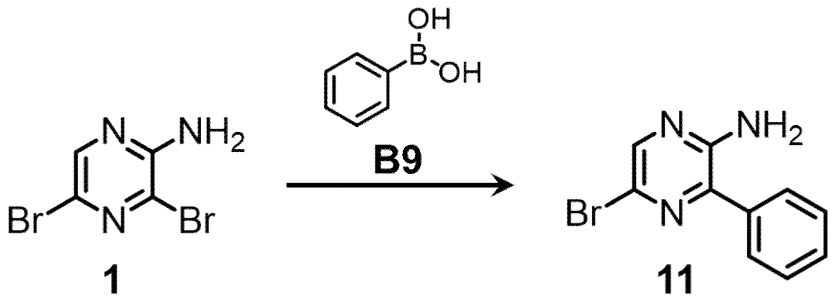

5-bromo-3-phenylpyrazin-2-amine 11
1H-NMR (500 MHz, ACETONE-D6) δ 8.05 (s, 1H), 7.75 (td, J = 4.7, 2.7 Hz, 2H), 7.53- 7.45 (m, 3H), 5.87 (s, 2H)
^{13}C-NMR (126 MHz, CHLOROFORM-D) δ 151.3, 142.9, 140.7, 135.9, 129.6, 129.2, 128.1, 127.1
HR-ESI-MS: *m/z*: [M+H]+ calcd for $C_{10}H_{9}{}^{79}Br_{1}N_{3}$, 249.99931; found, 249.99798
HR-ESI-MS: *m/z*: [M+H]+ calcd for $C_{10}H_{9}{}^{81}Br_{1}N_{3}$, 251.99954; found, 251.99594

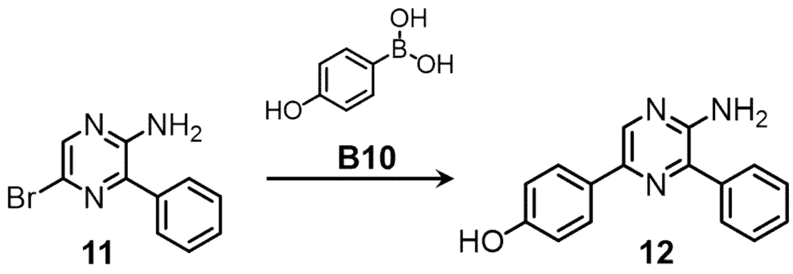

4-(5-amino-6-phenylpyrazin-2-yl)phenol 12

1H-NMR (500 MHz, ACETONE-D6) δ 8.45 (s, 1H), 7.90 (dd, J = 6.6, 2.0 Hz, 2H), 7.87 (d, J = 6.9 Hz, 2H), 7.55-7.51 (m, 2H), 7.47-7.44 (m, 1H), 6.93 (dd, J = 6.6, 2.0 Hz, 2H), 5.60 (s, 1H)

^{13}C-NMR (126 MHz, ACETONE-D6) δ 158.5, 152.2, 142.8, 139.3, 139.1, 137.8, 130.0, 129.5, 129.4, 129.2, 127.6, 116.4

HR-ESI-MS: *m/z*: [M+H]+ calcd for $C_{16}H_{14}N_3O_1$, 264.11360; found, 264.11369

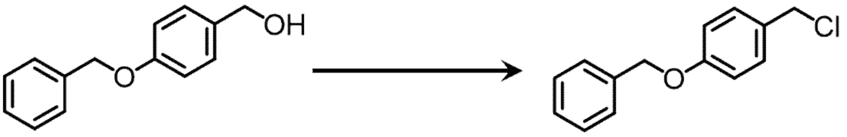

13 **14**

1-(benzyloxy)-4-(chloromethyl)benzene 14

4-Benzyloxybenzyl Alcohol **13** (2 g, 9.33 mmol) was dissolved in dry DCM (15 mL) and stirred at 0 °C under argon. Thionyl chloride (1.35 mL, 1.64 mmol) was added to the mixture, and the mixture was stirred for 2 hours at 0 °C. After the water was added to the mixture, the product was extracted with DCM (3 × 100 mL). The organic phase was dried over anhydrous sodium sulfate and evaporated. The achieved solid was washed by hexane to obtain compound **14** (1.6 g, 6.84 mmol, 74 %).

^{1}H-NMR (500 MHz, CHLOROFORM-D) δ 7.43-7.37 (m, 4H), 7.34-7.33 (m, 1H), 7.31 (dd, J = 6.6, 2.0 Hz, 2H), 6.95 (dd, J = 6.6, 2.0 Hz, 2H), 5.07 (S, 2H), 4.56 (S, 2H)
^{13}C-NMR (126 MHz, CHLOROFORM-D) δ 159.0, 136.9, 130.2, 130.1, 128.8, 128.2, 127.6, 115.2, 70.2, 46.4

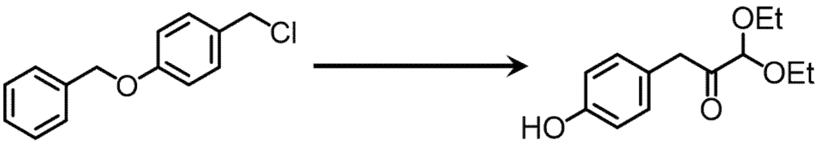

14 15

1,1-diethoxy-3-(4-hydroxyphenyl) propan-2-one 13

Mg turnings (666 mg, 27.50 mmol,) and 1,2-Dibromoethane (0.2 mL) were added to THF (10 ml) and stirred under argon for 20 minutes. The solution of 4-(Benzyloxy) benzyl Chloride **3** (1.6 g, 6.88 mmol) in THF (10 mL) was added and the mixture was stirred at room temperature for 30 minutes. Then the mixture was further refluxed for 1.5 hours to complete the reaction. The Grignard reagent was lowered to cool to room temperature and kept in an ice bath. Ethyl diethoxyacetate (1.29 ml, 7.28 mmol) was dissolved in THF (10 mL) under argon and cooled to –80 °C. The Grignard reagent was fallen in drops in the cooled flask over 15 min. The mixture was stirred at –78 °C for 2 hours. After the reaction was completed, 70 mL of water was added, and the product was extracted with ethyl acetate (3 × 100 mL). The organic phase was dried over anhydrous sodium sulfate and evaporated.

The obtained white oil substance was dissolved in MeOH (50 mL) and 10% Pd/C (190 mg) was added. And, the mixture was placed under H_2 gas and stirred for 24 hours. After the reaction was completed, the mixture was filtered through Celite and evaporated. The white oil substance was separated by column chromatography (hexane / ethyl acetate = 5 / 1 to 4 / 1) to obtain compound **12** (665 mg, 2.79 mol, 40%) as a colorless oil.

^{1}H-NMR (500 MHz, CHLOROFORM-D) δ 7.05 (dd, J = 11.5, 2.9 Hz, 2H), 6.75 (dd, J = 11.5, 3.4 Hz, 2H), 5.58 (S, 1H), 4.65 (S, 1H), 3.82 (S, 2H), 3.73-3.67 (m, 2H), 3.58-3.52 (m, 2H), 1.24 (t, J = 7.2 Hz, 6H)
^{13}C-NMR (126 MHz, CHLOROFORM-D) δ 204.5, 154.9, 130.9, 125.3, 115.6, 102.1, 63.5, 43.1, 15.2
HR-ESI-MS: *m/z*: [M+Na]+ calcd for $C_{13}H_{18}Na_{1}O_{4}$, 261.11141; found, 261.11028

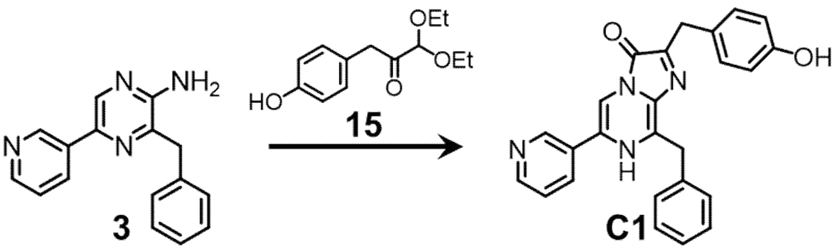

8-benzyl-2-(4-hydroxybenzyl)-6-(pyridin-3-yl)imidazo[1,2-a]pyrazin-3(7H)-one C1

Compound **2** (30 mg, 0.11 mmol) and compound **13** (40 mg, 0.17 mmol) were dissolved in ethanol at room temperature. 12M HCl aq (100 µL) was added to the mixture for 12 hours at 60°C under argon. The mixture was evaporated. The residue was separated by automated flash chromatography (Smart Flash EPCLC AI-580S, Universal Columns, chloroform /methanol = 99/1 to 15/85) to obtain analogue **C1** (20 mg, 0.048 mmol, 40 %, reddish—brown solid).

^{1}H-NMR (500 MHz, METHANOL-D4) δ 8.96 (s, 1H), 8.57 (dd, J = 4.9, 1.4 Hz, 1H), 8.21 (d, J = 8.0 Hz, 1H), 8.06 (s, 1H), 7.52 (dd, J = 8.0, 5.2 Hz, 1H), 7.39 (d, J = 7.4 Hz, 2H), 7.28 (t, J = 7.4 Hz, 2H), 7.22-7.19 (m, 1H), 7.13 (d, J = 8.6 Hz, 2H), 6.69 (d, J = 8.6 Hz, 2H), 4.41 (s, 2H), 4.07 (s, 2H)
HR-ESI-MS: *m/z*: [M+H]+ calcd for $C_{25}H_{21}N_4O_2$, 409.16635; found, 409.16645

Compounds **C2-C9** were prepared with a similar procedure to the preparation of **C1**.

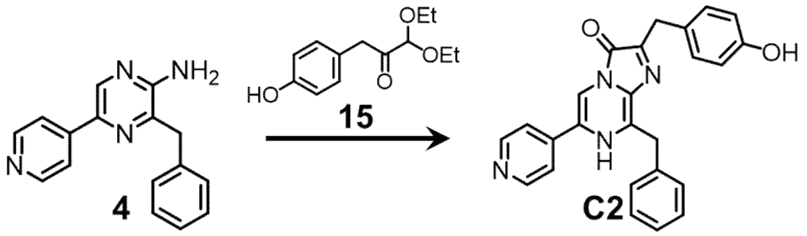

8-benzyl-2-(4-hydroxybenzyl)-6-(pyridin-4-yl)imidazo[1,2-a]pyrazin-3(7H)-one C2

^{1}H-NMR (500 MHz, METHANOL-D4) δ 9.17 (s, 1H), 8.90 (d, J = 6.9 Hz, 2H), 8.72 (d, J = 6.9 Hz, 2H), 7.42 (d, J = 6.9 Hz, 2H), 7.31-7.28 (m, 2H), 7.24-7.21 (m, 1H), 7.11 (dd, J = 11.5, 2.9 Hz, 2H), 6.71 (dd, J = 6.6, 2.0 Hz, 2H), 4.55 (s, 2H), 4.17 (s, 2H)

HR-ESI-MS: *m/z*: [M+H]+ calcd for $C_{25}H_{21}N_4O_2$, 409.16614; found, 409.16645

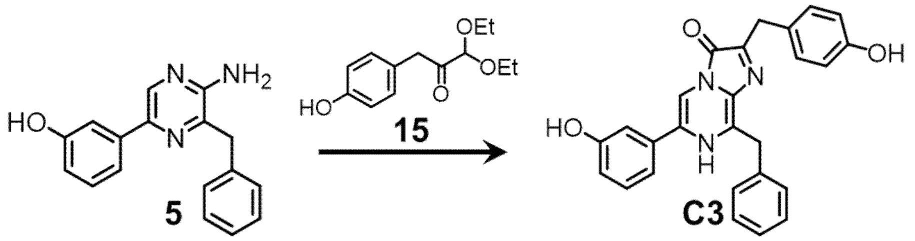

8-benzyl-2-(4-hydroxybenzyl)-6-(3-hydroxyphenyl)imidazo[1,2-a]pyrazin-3(7H)-one C3

^{1}H-NMR (500 MHz, METHANOL-D4) δ 8.46 (s, 1H), 7.40-7.28 (m, 8H), 7.23 (t, J = 7.2 Hz, 1H), 7.09 (d, J = 8.0 Hz, 2H), 6.91-6.89 (m, 1H), 6.72 (d, J = 8.6 Hz, 2H), 4.54 (s, 2H), 4.18 (s, 2H)

HR-ESI-MS: *m/z*: [M+H]+ calcd for $C_{26}H_{22}N_3O_3$, 424.16587; found, 424.16612

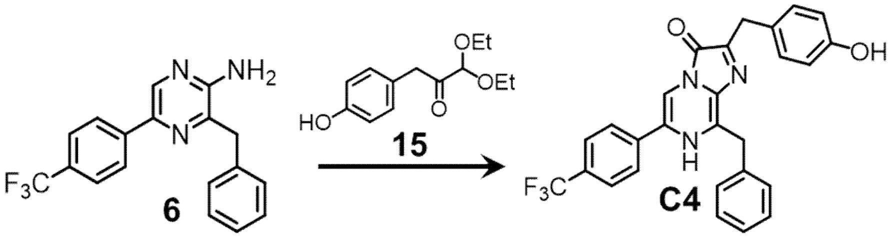

8-benzyl-2-(4-hydroxybenzyl)-6-(4-(trifluoromethyl)phenyl)imidazo[1,2-a]pyrazin-3(7H)-one C4

^{1}H-NMR (500 MHz, METHANOL-D4) δ 7.91 (s, 1H), 7.84 (d, J = 7.4 Hz, 2H), 7.67 (d, J = 8.0 Hz, 2H), 7.29 (d, J = 7.4 Hz, 2H), 7.18 (t, J = 7.4 Hz, 2H), 7.12 (t, J = 7.4 Hz, 1H), 7.04 (d, J = 8.6 Hz, 2H), 6.59 (d, J = 8.0 Hz, 2H), 4.32 (s, 2H), 3.97 (s, 2H)

HR-ESI-MS: *m/z*: [M+H]+ calcd for $C_{27}H_{21}F_3N_3O_2$, 476.15722; found, 476.15859

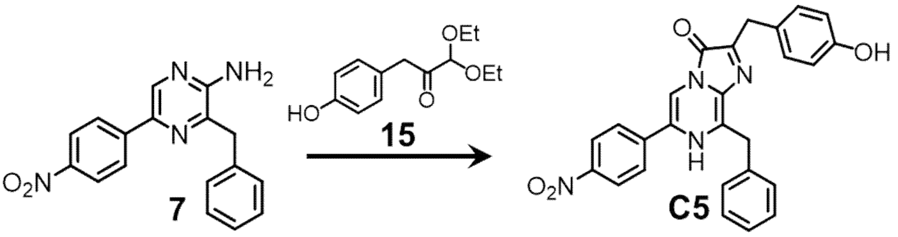

8-benzyl-2-(4-hydroxybenzyl)-6-(4-nitrophenyl)imidazo[1,2-a]pyrazin-3(7H)-one C5

^{1}H-NMR (500 MHz, METHANOL-D4) δ 8.30 (d, J = 9.2 Hz, 2H), 8.19 (s, 1H), 8.04 (d, J = 8.0 Hz, 2H), 7.38 (d, J = 6.9 Hz, 2H), 7.28-7.25 (m, 2H), 7.21-7.19 (m, 1H), 7.11 (d, J = 8.6 Hz, 2H), 6.68 (d, J = 8.6 Hz, 2H), 4.41 (s, 2H), 4.06 (s, 2H)

HR-ESI-MS: *m/z*: [M+H]+ calcd for $C_{26}H_{21}N_4O_4$, 453.15907; found, 453.15628

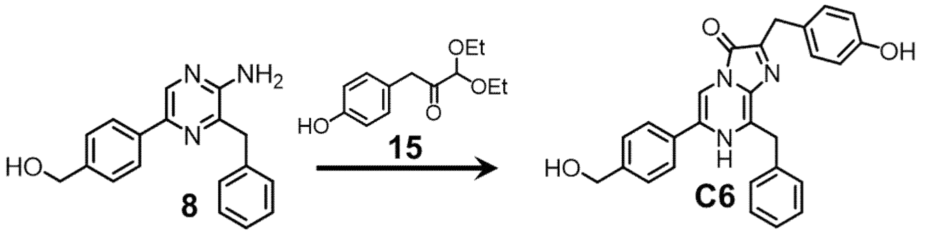

8-benzyl-2-(4-hydroxybenzyl)-6-(4-(hydroxymethyl)phenyl)imidazo[1,2-a]pyrazin-3(7H)-one C6

^{1}H-NMR (500 MHz, METHANOL-D4) δ 7.71 (s, 1H), 7.63 (d, J = 7.4 Hz, 2H), 7.46 (d, J = 8.6 Hz, 2H), 7.39 (d, J = 8.0 Hz, 2H), 7.29 (t, J = 7.4 Hz, 2H), 7.22 (t, J = 7.4 Hz, 1H), 7.15 (d, J = 8.0 Hz, 2H), 6.69 (d, J = 8.6 Hz, 3H), 4.65 (s, 2H), 4.41 (s, 2H), 4.07 (s, 2H)

HR-ESI-MS: *m/z*: [M+H]+ calcd for $C_{27}H_{24}N_3O_3$, 438.18124; found, 438.18177

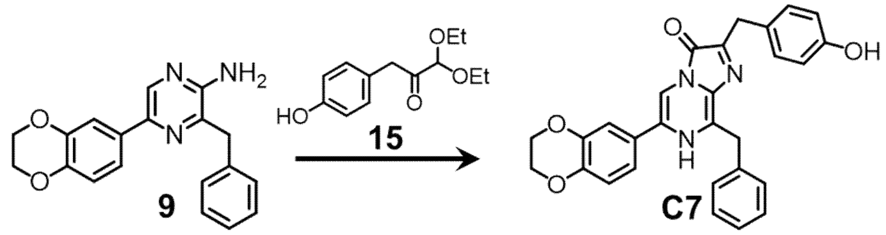

8-benzyl-6-(2,3-dihydrobenzo[b][1,4]dioxin-6-yl)-2-(4-hydroxybenzyl)imidazo[1,2-a]pyrazin-3(7H)-one C7

^{1}H-NMR (500 MHz, METHANOL-D4) δ 7.71 (s, 1H), 7.63 (d, J = 7.4 Hz, 2H), 7.46 (d, J = 8.6 Hz, 2H), 7.39 (d, J = 8.0 Hz, 2H), 7.29 (t, J = 7.4 Hz, 2H), 7.22 (t, J = 7.4 Hz, 1H), 7.15 (d, J = 8.0 Hz, 2H), 6.69 (d, J = 8.6 Hz, 3H), 4.65 (s, 2H), 4.41 (s, 2H), 4.07 (s, 2H)

HR-ESI-MS: *m/z*: [M+H]+ calcd for $C_{27}H_{24}N_3O_3$, 438.18124; found, 438.18177

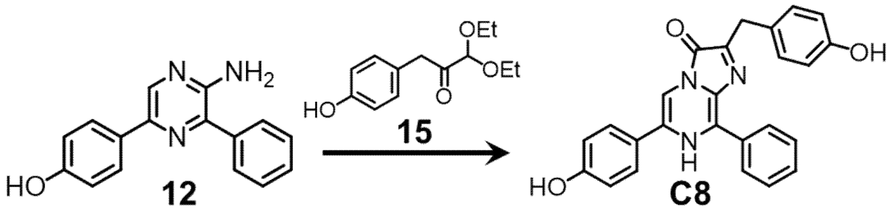

2-(4-hydroxybenzyl)-6-(4-hydroxyphenyl)-8-phenylimidazo[1,2-a]pyrazin-3(7H)-one C8

^{1}H-NMR (500 MHz, METHANOL-D4) δ 8.01 (d, J = 5.2 Hz, 2H), 7.65 (s, 1H), 7.60-7.55 (m, 3H), 7.13 (d, J = 8.6 Hz, 2H), 6.90 (dd, J = 6.6, 2.0 Hz, 2H), 6.66 (dd, J = 6.6, 2.0 Hz, 2H), 4.03 (s, 2H) HR-ESI-MS: *m/z*: [M+H]+ calcd for $C_{25}H_{20}N_3O_3$, 410.14906; found, 410.15047

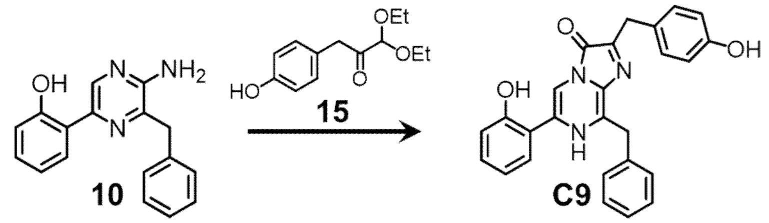

8-benzyl-2-(4-hydroxybenzyl)-6-(2-hydroxyphenyl)imidazo[1,2-a]pyrazin-3(7H)-one C9

^{1}H-NMR (500 MHz, METHANOL-D4) δ 7.48-7.57 (s, 1H), 7.38 (d, J = 7.4 Hz, 2H), 7.29 (t, J = 7.4 Hz, 2H), 7.23 (t, J = 7.2 Hz, 2H), 7.13 (d, J = 8.6 Hz, 2H), 6.89 (t, J = 7.7 Hz, 1H), 6.85 (d, J = 9.2 Hz, 1H), 6.68 (d, J = 8.6 Hz, 2H), 4.40 (s, 2H), 4.07 (s, 2H)

HR-ESI-MS: *m/z*: [M+H]+ calcd for $C_{26}H_{22}N_3O_3$, 424.16630; found, 424.16612
